# Enhancing help-seeking behaviour among men who have sex with men at risk for sexually transmitted infections: the syn.bas.in randomised controlled trial

**DOI:** 10.1136/sextrans-2020-054438

**Published:** 2020-07-31

**Authors:** Roeland Christiaan Alfons Achterbergh, Martijn S van Rooijen, Wim van den Brink, Anders Boyd, Henry John Christiaan de Vries

**Affiliations:** 1 STI Outpatient Clinic, Infectious Diseases Department, Public Health Sevice of Amsterdam, Amsterdam, North Holland, The Netherlands; 2 Department of Psychiatry, Amsterdam UMC Location AMC, Amsterdam, North Holland, The Netherlands; 3 Department of Infectious Diseases Research and Prevention, Public Health Service of Amsterdam, Amsterdam, Noord-Holland, The Netherlands; 4 Stichting HIV Monitoring, Amsterdam, Noord-Holland, The Netherlands; 5 Department of Dermatology, Amsterdam Institute for Infection and Immunity (AI&II), location Academic Medical Centre, Amsterdam UMC, University of Amsterdam, Amsterdam, North Holland, The Netherlands

**Keywords:** homosexuality, sexual health, substance misuse

## Abstract

**Objectives:**

Men who have sex with men (MSM) are at increased risk for STIs and mental disorders. Syndemic theory holds that psychosocial issues co-occur and interact, and thus increase sexual risk behaviour. Psychosocial issue identification, referral and management might reduce risk behaviour.

**Methods:**

In the syndemic-based intervention study, an open-label randomised controlled trial, MSM were enrolled at the STI outpatient clinic of the Public Health Service of Amsterdam. We screened participants using validated questionnaires on the following problem domains: alcohol and substance use, sexual compulsivity, anxiety, depression, attention deficit hyperactivity disorder, alexithymia, intimate partner violence and childhood sexual abuse. Individuals were randomly assigned (1:1) to receive either tailored, face-to-face feedback and help-seeking advice on mental health screening, or no feedback and no help-seeking advice. Participants were followed trimonthly for a year. The primary outcomes were self-reported and confirmed help-seeking behaviour.

**Results:**

We included 155 MSM: 76 in the intervention group and 79 in the control group. At inclusion, 128 participants (83.1%) scored positive in at least one problem domain. We found no significant differences in self-reported or confirmed help-seeking behaviour between the intervention and the control group: 41% vs 29% (p=0.14) and 28% vs 22% (p=0.44), respectively. There were also no differences in STI incidence and condomless anal sex acts between the two groups.

**Conclusion:**

Screening showed high prevalence of problems related to mental health and substance use, while tailored feedback, advice and referral did not significantly increase help-seeking behaviour. Other interventions are needed to tackle the high burden of mental disorders among MSM.

**Trial registration number:**

NCT02859935.

## Introduction

Men who have sex with men (MSM) constitute a risk group for STIs and are at increased risk of poor mental health and substance use compared with the general population.^1 2^ The co-occurrence of afflictions that interact synergistically and contribute to an excess burden of disease is called a syndemic or synergy.^3^ The syndemic theory holds that multiple adverse conditions frequently co-occur, interact and mutually reinforce each other, thereby increasing risk behaviours and co-occurring diseases.

Ron Stall was the first to acknowledge that syndemicity could potentially explain the high STI and HIV incidence found in an MSM core group.^4^ This idea was further explored by others. For example, in some studies among MSM, an increasing number of mental health-related problem domains (including depression, substance use problems, childhood abuse, intimate partner violence) were shown to be associated with increased sexual risk behaviour, such as a higher number of condomless anal sex (CAS) acts and an increased prevalence of STI or HIV infection.^4–7^ Furthermore, some studies found that HIV-positive MSM and MSM using pre-exposure prophylaxis (PrEP) have an increased risk of non-adherence to medication, increased number of sex partners and increased CAS acts when substance use and depression are present, thereby increasing the risk of onwards HIV acquisition and transmission.^8–11^

While studies have found different patterns of effects, assessing and managing co-occurring mental health-related problem domains may decrease high-risk behaviours.^12 13^ Several studies combining mental and sexual health among MSM have focused on increasing adherence to antiretroviral therapy (ART) or decreasing CAS by using cognitive behavioural therapy.^9 14 15^ However, these studies focused on a single issue, while the syndemic theory emphasises the importance of a holistic approach involving multiple issues.

Previously, we initiated a cohort for MSM at increased risk of STI: the MS2 cohort, offering STI screening four times a year. Substance use and STI positivity remained high at 25%, despite motivational interviewing and counselling.^16^ Recently, many promising behavioural interventions addressing psychosocial syndemic and HIV-related health behaviours in MSM have emerged.^17^ However, although STI clinics offer opportunities to reach out to affected populations, they are not always equipped to address mental health-related issues. Constrained by the limitations of our setting, we decided to develop a health intervention directed at help-seeking behaviours and an increase in referrals to settings dedicated to the treatment of mental health-related problems.

In an open-label, randomised controlled trial (RCT), we assessed if screening for mental health-related problems and tailored feedback might increase help-seeking behaviour and decrease risk behaviour in MSM at high risk for STIs. To determine whether the intervention affects psychosocial domains, sexual behaviour, substance use and help-seeking behaviour over time, we also examined the longitudinal changes in these end points and compared them between screening arms.

## Methods

### Study design and participants

This randomised-controlled, parallel-group, open-label, superiority trial was conducted at the STI outpatient clinic of the Public Health Service (PHS) of Amsterdam.

The study design and protocol of this syndemic-based intervention (syn.bas.in) have been published previously.^18^ We recruited MSM from the MS2 cohort, which included MSM with high-risk behaviour who are screened trimonthly for STIs.^16^ Eligible participants for the syn.bas.in study were self-identified MSM, aged 18 years or above and showing high-risk sexual behaviour within the last 24 months (for HIV-negative MSM: having had two or more STI diagnoses or postexposure prophylaxis treatment; for HIV-positive MSM: having had one or more STI diagnosis). Recruitment occurred between 1 September 2016 and 1 September 2017. Data collection ended at 1 September 2018. The study was funded by the PHS of Amsterdam (project 2372394). Following the recommendations of Michie *et al*,^19^ a theoretical framework for our help-seeking intervention is presented in online supplementary appendix 1.

10.1136/sextrans-2020-054438.supp1 Supplementary data

### Randomisation

We randomly assigned participants (1:1) to an intervention and control group, using a computer-generated randomisation list with variable permuted block sizes (four, six and eight) after informed consent was obtained. Randomisation was not stratified. The nature of the intervention required participants and study healthcare providers to be aware of group allocation, but they did not know the allocation until participants provided informed consent and were enrolled in the study. A table assessing risk of bias is provided in online supplementary appendix 2.

### Procedures

A detailed flow chart of the study design can be found in online supplementary appendix 3. During an MS2 cohort visit, the study was explained by study personnel, written informed consent was obtained and participants were randomised. This visit was defined as the inclusion visit (T0). All participants were asked to attend the outpatient clinic trimonthly for 1 year during: an intervention/control allocation visit (T3) and three follow-up visits (T6, T9 and T12). During all visits, participants received standard care, including STI screening, and a motivational interviewing-based sex-counselling session.

After inclusion (T0), participants were invited to fill out a set of questionnaires sent by email using computer-assisted self-interviews (CASI) representing several mental health-related domains. Previous research has shown associations of HIV positivity and/or risk behaviour with several syndemic problem domains, including sexual compulsivity, substance or alcohol use, depression, partner violence, childhood sexual abuse and discrimination.^18^ In addition, people with ADHD may engage in risky sexual practices due to problems with impulsivity. Alexithymia is an important factor associated with depression or anxiety, is often diagnosed in persons with substance abuse and may explain the lack of awareness of anxiety and depression in some addicted individuals. We used the following questionnaires: (1) the Alcohol Use Dependency Identification Test^18^; (2) Drug Use Dependency Identification Test^18^; (3) the Sexual Compulsivity Scale^18^; (4) the Hospital Anxiety and Depression Scale^18^; (5) intimate partner violence in the past 5 years (yes/no); (6) childhood sexual abuse before age ≤16 years (yes/no)^4–6^; (7) the Adult ADHD Self-Report Scale^18^ and (8) the Toronto Alexithymia Scale.^18^

One week before the subsequent intervention visit (T3), participants in the intervention group received a second round of questionnaires on mental health-related domains, the results of this round were used to provide updated feedback during the intervention. At the intervention visit (T3), the results of all questionnaires were discussed to provide participants with an accurate insight in potential mental health-related issues and their possible contribution to risk behaviour. Using tailored feedback, motivational interviewing and help-seeking advice, willingness to seek help for mental health or addiction treatment was evoked.

Participants in the control group did not receive the results of their questionnaires nor tailored feedback or help-seeking advice.

Participants in both groups were invited for follow-up visits with standard care (T6, T9 and T12) during which help-seeking behaviour for mental health or addiction was monitored. At the final visit (T12), all participants of both study groups again received a round of questionnaires on mental health-related domains, and were allowed to discuss these results for ethical reasons.

All study healthcare providers received training by mental health and addiction care professionals to appropriately address problem domains. To reduce barriers and improve help-seeking behaviour, mental healthcare and substance use services at the STI clinic were established, whereby monthly intake consultations were available free-of-charge (co-located care). Participants of both study groups had access to co-located care.

At every study visit, participants filled out a questionnaire on sexual behaviour and substance use in the preceding 3 months using CASI. One reminder was sent if questionnaires were not returned. Participants received two reminders to visit the STI clinic if they did not appear for a final visit.

### Outcomes

Our primary end point was the proportion of participants who sought help from a mental healthcare or addiction treatment service during the study up until T12. The primary end point was assessed by self-reports and confirmation with the co-located care or the attended clinic (if care was sought outside the STI clinic).

Secondary end points were STI incidence and sexual behaviour, for example, number of partners, number of anal sex acts and number of CAS acts with casual partners. STI and HIV testing were performed according to routine STI clinic protocols at every visit.^20^ In case of symptoms suggestive of an STI or partner notification, additional STI testing was allowed between regular study visits. Results from these additional visits were included in analysis. Routine STI testing, including HCV testing were performed as described previously.^21 22^

### Statistical analyses

The sample size was calculated to detect a difference of 18% in help-seeking behaviour: 7% in the control group (as estimated from Amsterdam health monitor^23^) vs 25% in the intervention group. With a significance level of α=5% and a minimal statistical power of 80%, 64 participants per group would be needed to determine a statistically significant result. Assuming 10% loss to follow-up, a minimum of 142 participants was required. Therefore, we aimed to recruit 150 participants.

For the primary end point, we calculated differences in (self-reported and confirmed) help-seeking behaviour between the intervention and the control group using a logistic regression model. Help-seeking behaviour (yes/no) was used as the dependent variable, and treatment group (intervention/control) as the independent variable. We conducted a modified intention-to-treat (mITT) analysis, in which we included participants who completed the questionnaire of follow-up visit T6, T9 or T12. In the per-protocol (PP) analysis, we only included participants who completed the study questionnaire at T12.

STI incidence rates (IRs) per 100 person-years (py) were calculated as the total number of visits with a bacterial STI divided by the py of observation and we included repeat STI infections over time. We calculated 95% CI for IR using quadratic approximation method or exact Poisson method. We evaluated change in STI incidence over visits using an exponential survival model with gamma-distributed shared frailty, to account for between-subject variability. We compared overall IRs between groups using p values calculated from the Wald χ^2^ test.

We examined longitudinal changes for psychosocial domains (T0 and T12) and sexual behaviour and substance use (T3, T6, T9 and T12) during follow-up. For each outcome, we used mixed-effect logistic regression including study group (intervention and control), study visit and the interaction between the two. A random intercept was added to account for baseline differences between individuals. We tested for changes over time using an analysis of variance-type joint test, nested within group.

Furthermore, we performed univariable logistic regression analysis to identify variables associated with confirmed help-seeking behaviour. Variables associated with help-seeking at p<0.15 were included in a multivariable model using backward selection. Due to multicollinearity between mental health-related problem domains, we only included the total number of different mental health-related problem domains as a variable. Study group was forced into the model.

Statistical analyses were performed with STATA Intercooled V.13.1 (STATA, College Station, Texas, USA). Significance was defined as a p value <0.05.

## Results

**Figure 1:**
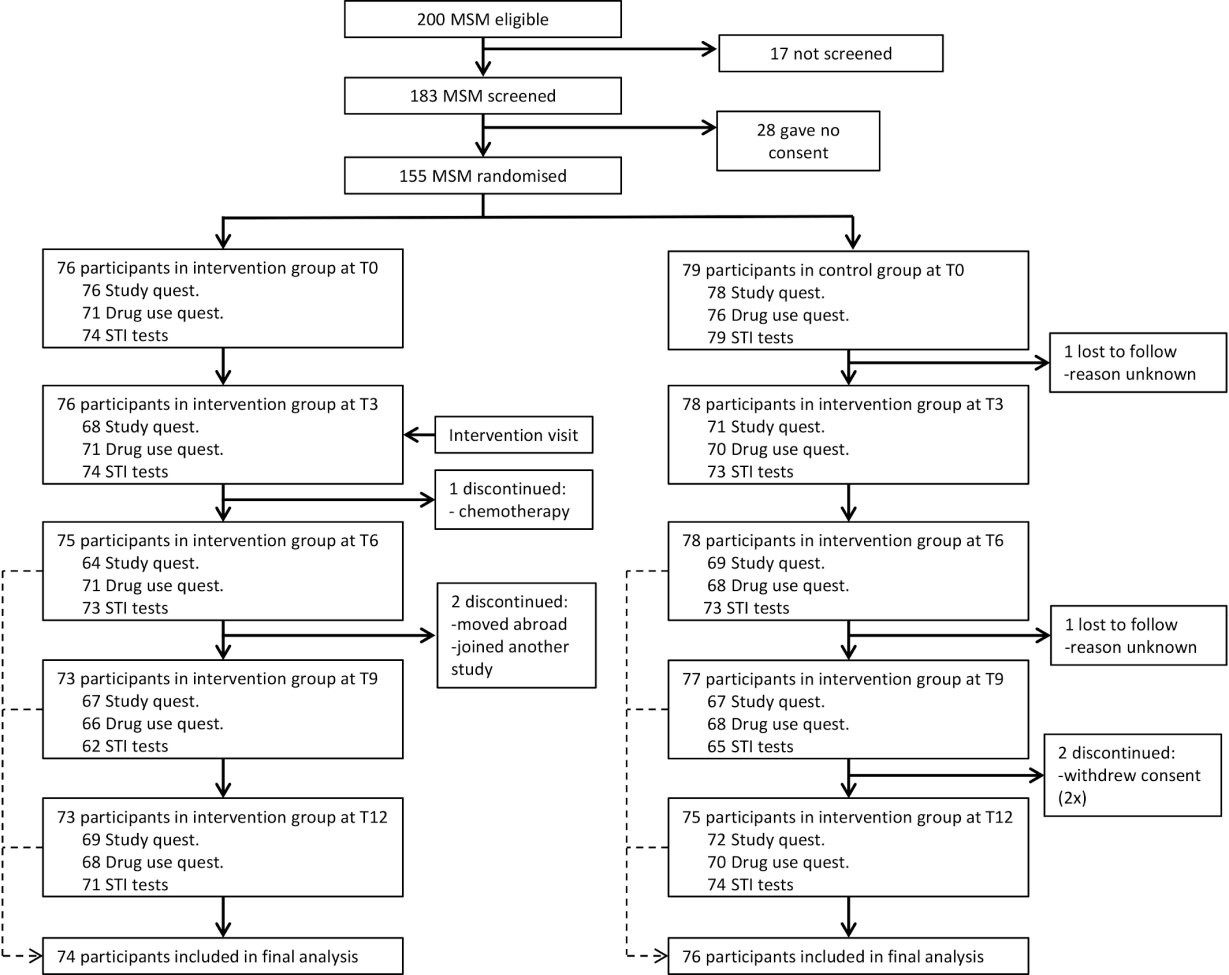
Enrolment and follow-up of study participants of the syndemic-based intervention (syn.bas.in) study at the STI outpatient clinic in Amsterdam, 2016–2018.

Between 1 September 2016 and 1 September 2017, 200 men were identified as eligible. Seventeen were not screened and 28 did not provide consent. Thus, 155 were enrolled in the trial (figure 1). Participants who were not screened did not differ in age or ethnicity from those who were screened (p>0.05). Participants who did not provide consent also did not differ in age or ethnicity form those who provided consent (p>0.05). We randomised 76 MSM to the intervention group and 79 MSM to the control group.

Characteristics at inclusion visit are described in table 1. Median age was 43 (IQR 34–51), 66% were Dutch, 66% were HIV-positive and median number of sex partners in the preceding 3 months was 14 (IQR 6–25). Thirty-six of the 155 participants (23%) had a bacterial STI and 1 of the 51 participants (2%) who had previously tested HIV negative was newly diagnosed HIV positive at baseline. At inclusion, only 26 (17%) participants scored negative on all mental health-related problem domains, 45 (30%) scored positive on one domain, 29 (19%) on two domains, 20 (13%) on three domains, 20 (13%) on four domains and 14 (9%) on five or more domains.

**Table 1:** Characteristics of 155 men who have sex with men at study inclusion of the syndemic-based intervention (syn.bas.in) study at the STI outpatient clinic in Amsterdam, 2016–2017

	Intervention (n=76)	Control (n=79)
Demographics		
Age (years)		
Median (IQR)	46 (35–52)	42 (33–49)
Country of origin		
The Netherlands	54 (71%)	49 (62%)
Non-Dutch high-income country	7 (9%)	13 (16%)
Other	15 (20%)	17 (22%)
Educational level		
Low and middle	15 (20%)	22 (28%)
High	61 (80%)	57 (72%)
Sexual health/behaviour characteristics		
Number of sex partners (3 months)		
Median (IQR)	15 (6.5–24.5)	13 (6–26)
Condomless anal sex (CAS)		
No CAS	13 (17%)	12 (15%)
Insertive CAS only	4 (5%)	4 (5%)
Receptive CAS only	7 (9%)	12 (15%)
Insertive and receptive CAS	52 (68%)	50 (64%)
Number of CAS acts with casual partners (3 months)		
Median (IQR)	5.5 (2–16.5)	5 (1–18)
HIV status		
HIV-negative	25 (33%)	27 (34%)
HIV-positive	51 (67%)	52 (66%)
STI		
Chlamydia	3 (4%)	11 (14%)
Gonorrhoea	8 (11%)	12 (15%)
Syphilis (recent, stage 1 or 2)	5 (7%)	3 (4%)
New HIV diagnosis	0/24 (0%)	1/27 (4%)
Hepatitis C	0	0
Any bacterial STI (chlamydia, gonorrhoea or syphilis)	14 (19%)	22 (28%)
Substance use during sex in the past 3 months*		
Alcohol	24 (33%)	26 (34%)
Amphetamine	17 (24%)	20 (26%)
Cannabis	20 (28%)	25 (33%
Cocaine	16 (22%)	14 (18%)
Erectile dysfunction drugs	53 (74%)	49 (64%)
GHB/GBL	31 (43%)	34 (45%)
Ketamine	9 (13%)	19 (25%)
Methamphetamine	9 (13%)	12 (16%)
Mephedrone	5 (7%)	5 (7%)
Nitrites	52 (72%)	53 (70%)
XTC/MDMA	34 (47%)	33 (43%)
Other	1 (1%)	0 (0%)
Tobacco (in general)	24 (33%)	29 (38%)
Mental health-related problem domains†		
Alcohol use disorder		
AUDIT score ≥8	19 (25%)	28 (36%)
Drug use disorder		
DUDIT score ≥8	36 (47%)	40 (51%)
Sexual compulsivity		
SCS score ≥24	17 (22%)	11 (14%)
Anxiety disorder or depression		
HADS depression score ≥8	11 (14%)	15 (19%)
HADS anxiety score ≥8	21 (28%)	26 (33%)
ADHD		
Score ≥4	8 (11%)	6 (8%)
Alexithymia		
Score 52–62 (possible indication)	20 (26%)	12 (15%)
Score >62 (indication)	12 (16%)	11 (14%)
Partner violence (yes)	4 (5%)	8 (10%)
Childhood abuse (yes)	9 (12%)	8 (10%)
Discrimination		
Median (IQR)	13 (11–17)	14 (10–17)
Score indicates positive on at least one mental health-related issue	59 (78%)	69 (88%)
Self-reported psychosocial care preceding year	23 (30%)	25 (32%)

*Four missing in the intervention group, three in the control group.

†One missing in the control group.

ADHD, attention deficit hyperactivity disorder; AUDIT, Alcohol Use Dependency Identification Test; DUDIT, Drug Use Dependency Identification Test; GBL, gamma-butyrolactone; GHB, gamma-hydroxybutyric acid; HADS, Hospital Anxiety and Depression Scale; SCS, Sexual Compulsivity Scale; XTC, ecstasy.

Overall, 74 of the 76 (97%) participants in the intervention group and 76 of the 79 (96%) participants in the control group had at least one follow-up visit with completed questionnaires after the intended intervention visit (T3) (figure 1). Median follow-up time in the intervention group was 1.0 years (IQR (1.0–1.1), in total 81.0 py) and in the control group 1.0 years (IQR (1.0–1.1), in total 83.8 years), totalling 164.9 py.

In mITT analysis, self-reported help-seeking behaviour during follow-up was observed in 30/74 (40.5%, 95% CI 29.3% to 52.6%) participants in the intervention group and in 22/76 (28.9%, 95% CI 19.1% to 40.5%) participants in the control group (p=0.136, table 2). Confirmed help-seeking behaviour was observed in 20/72 (27.8%, 95% CI 17.9% to 39.6%) in the intervention group vs 16/72 (22.2%, 95% CI 13.3% to 33.6%) in the control group (p=0.441). In PP analysis, self-reported help-seeking behaviour was observed in 29/69 (42.0%, 95% CI 30.2% to 54.5%) participants of the intervention group and 20/72 (27.8%, 95% CI 17.9% to 39.6%) participants of the control group (p=0.076). These proportions were lower with confirmed help-seeking behaviour: 19/67 (28.4%, 95% CI 18.0% to 40.7%) in the intervention group and 14/68 (20.6%, 95% CI 11.7% to 32.1%) in the control group (p=0.294). Six individuals did not consent for their help-seeking behaviour to be confirmed and were considered as missing.

**Table 2:** Characteristics of 150 men who have sex with men in the syndemic-based intervention (syn.bas.in) study at the STI outpatient clinic in Amsterdam, 2017–2018

	Intervention	Control	P value
Self-reported help-seeking behaviour			
mITT analysis	30/74 (41%)	22/76 (29%)	0.136
PP analysis	29/69 (42%)	20/72 (28%)	0.076
Confirmed help-seeking behaviour*			
mITT analysis	20/72 (28%)	16/72 (22%)	0.441
PP analysis	19/67 (28%)	14/68 (21%)	0.294
STI rates (n/100 person-years)			
Chlamydia any site	47.3 (33.7 to 66.4)	46.3 (33.1 to 64.6)	0.926
Pharyngeal	4.9 (1.9 to 13.2)	1.2 (0.2 to 8.5)	0.204
Urethral	13.5 (7.1 to 25.6)	21.5 (12.7 to 36.2)	0.269
Anorectal	33.3 (21.0 to 52.8)	30.3 (19.2 to 47.9)	0.777
Of which LGV	3.7 (1.2 to 11.5)	6.0 (2.5 to 14.3)	0.515
Gonorrhoea any site	59.2 (44.6 to 78.6)	52.4 (39.0 to 70.5)	0.559
Pharyngeal	23.4 (15.0 to 36.8)	20.2 (12.6 to 32.6)	0.662
Urethral	9.9 (5.0 to 20.4)	11.9 (6.2 to 22.9)	0.711
Anorectal	48.2 (34.8 to 66.8)	44.1 (31.6 to 61.6)	0.709
Syphilis (recent, stadium 1 or 2)	6.2 (2.6 to 14.8)	13.1 (7.3 to 23.7)	0.163
HIV	0	0	/
Hepatitis C	0	1.2 (0.2 to 8.4)	0.995
Any bacterial STI	105.1 (72.6 to 137.3)	104.9 (83.9 to 131.2)	0.991

*Unable to obtain six for whom informed consent was not obtained, two in the intervention group and four in the control group.

LGV, lymphogranuloma venereum; mITT, modified intention-to-treat; PP, per-protocol.

**Figure 2:**
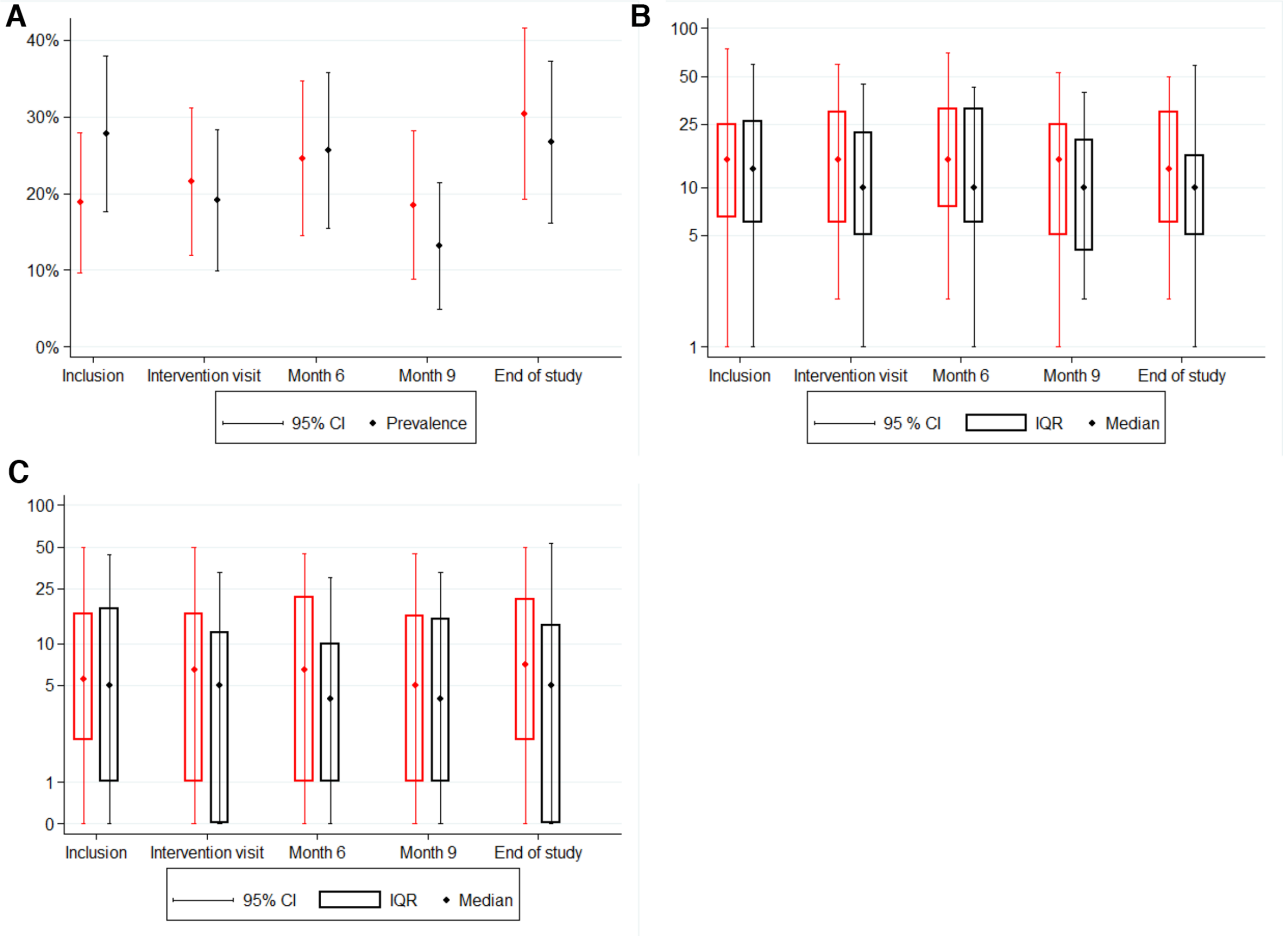
Sexual behaviour over time by intervention and control group among men who have sex with men in the syndemic-based intervention (syn.bas.in) study at the STI outpatient clinic in Amsterdam, 2016–2018. (A) Number of partners over time (no difference between intervention (red) and control (black) (p=0.546). (B) Number of condomless anal sex acts with casual partners (no difference between intervention (red) and control (black) (p =0.494)). STI prevalence (no difference between intervention(red) and control (black) (p =0.914)).

STI incidence was 105.1/100 py (95% CI 72.6 to 137.3/100 py) in the intervention group vs 104.9/100 py (95% CI 83.9 to 131.2/100 py) in the control group (p=0.99). We also found no significant differences between intervention and controls over time for number of sex partners (figure 2A) or number of CAS acts with casual partners (figure 2B) or STI incidence (figure 2 C).

We found a significant decrease for drug-related problems (49.4% at T0 vs 36.9% at T12, p=0.005) and sexual compulsivity (18.1% at T0 vs 9.2% at T12, p=0.03) over time, but there were no between-group differences (p>0.05). We found no significant differences in proportion with substance use over time or between groups (online supplementary appendix 4). No serious adverse events due to the intervention were reported during follow-up.

Having a drug-related problem, sexual compulsivity, depression, anxiety, AHDH and childhood sexual abuse were all associated with confirmed help-seeking behaviour in univariable analysis (online supplementary appendix 5). Having more mental health disorders increased the odds of seeking help in multivariable analysis (adjusted OR (aOR) per disorder 1.9, 95% CI 1.4 to 205), while not being born in The Netherlands (aOR 0.4, 95% CI 0.1 to 1.0) was associated with less help-seeking behaviour.

## Discussion

Our results show that, at baseline, >80% of participants scored positive on at least one mental health-related domain, with roughly 20% of all participants having at least four mental health-related domains. The intervention comprising screening, tailored advice and referral to a (co-located) mental health or addiction service did not lead to more frequent help-seeking behaviour, be it self-reported or confirmed. Of those scoring positive on any mental health-related issue, 38.4% reported help-seeking behaviour. This is comparable to individuals with a mental health issue in the general Dutch population (33.8%).^24^ MSM with a higher number of mental health problems were more likely to engage in help-seeking behaviour. We also observed a high incidence of STIs, frequent CAS and frequent substance use over time without any significant differences between the intervention and control group.

Although we failed to observe a significant effect of the intervention, a 10% higher proportion of individuals in the intervention group sought mental care, compared with the control group. Of the few RCTs evaluating mental health screening and tailored advice, one conducted in postdeployment UK military personnel and another among elite athletes, none observed an effect on help-seeking behaviour.^25 26^ Even though our syndemic-based approach failed to reduce help-seeking behaviour or STI incidence, the frequent concomitance of mental health disparities in MSM warrants the use of an holistic approach.^3^

One potential reason why no significant effect on help-seeking behaviour was observed could be participants’ lack of interest in engaging with mental health or addiction treatment services or downplaying of mental problems. A report from STI clinics in Vancouver, Canada reported barriers to help-seeking behaviour for unmet mental health needs.^27^ They also mentioned downplaying mental health problems, a desire for self-management and additionally shame and being unable to find or afford services. The latter two reasons would not be considered issues in our study due to co-located care. Another reason for the absence of a significant effect could be a lack of experience with tailored referrals among health providers of the STI clinic. Yet, all healthcare providers were experienced in motivational interviewing, and received additional training to address mental health and substance use-related issues. On average, each healthcare provider saw 11 participants with a need for referral. It is arguable that additional experience could benefit successful referrals. Although not significant, we did record a higher ratio in help-seeking behaviour in the intervention group. Therefore, this study might have been underpowered, and larger groups might reveal significant effect in future studies.

Feedback from study healthcare personnel indicated that participants in both groups appreciated the regular visits with a trusted healthcare provider. The study itself allowed them to discuss hard-to-address mental health-related issues. These signals indicate the need to address the syndemic concept in a sexual health setting. Interestingly, we found a significant decrease of sexual compulsivity and drug-related problems over time in both groups. Similar findings were observed in a recent analysis of our prospective Amsterdam PrEP demonstration project, in which we also offered trimonthly motivational-based counselling regarding sexual health plus comprehensive assessments of mental health, substance use and sexual behaviour.^28^

We did not find a difference in sexual behaviour or STI incidence between the intervention and control groups. The overall STI incidence was particularly high in this study (105.0/100 py) compared with MSM in the Amsterdam Cohort Studies in 2016 (19.6/100 py),^29^ which aims to study STI risk in the general MSM population of Amsterdam. PrEP was not registered in Europe at the beginning of the study, and we diagnosed one HIV infection at baseline, stressing the importance of PrEP in any future intervention focused on MSM at increased risk for STI.

Strengths of our study are the use of both self-reported and confirmed help-seeking behaviour, since participants might tend to provide more socially desirable responses as stressed by the large differences observed between self-reported and confirmed help-seeking behaviour. For the same reason, we also recorded laboratory-confirmed STIs, and not self-reported STIs. Furthermore, at 4.5% within 1 year, we report very low rates of loss to follow-up compared with other mental health interventions among MSM.^9 14 15^ It shows that STI clinics are feasible locations to retain MSM when identifying and discussing mental health-related issues. Lastly, the option for co-located care instead of referral to an external hospital or care setting, likely lowered lost to follow-up.

Limitations are first, the preference for referrals to external care settings over co-located care was much higher than expected. Limited by human resources, co-located care was available only once a month. This might have been too infrequent to have an effect. Integrating mental and sexual health services might be of benefit to increase successful referrals. Second, the study consisted primarily of highly educated MSM with a Dutch/Western background, generalisability of our findings is thus limited. Third, the validated questionnaires are screening tools and are not developed for diagnosing mental disorders. Lastly, help-seeking behaviour was only measured in general, and not specific to a given mental health disorder.

In conclusion, screening for mental health-related issues and providing tailored advice and referral to mental health and addiction treatment services did not increase help-seeking behaviour among MSM at high risk for STI. Considering the disturbingly high prevalence of mental health-related problem domains and high STI incidence, other interventions improving mental and sexual health and substance use among MSM in sexual health settings are needed.

**Key messages**

- An intervention including screening for mental disorders plus tailored advice did not result in a significant increase in self-reported or confirmed help-seeking behaviour in men who have sex with men at increased risk for STIs.
- At baseline, >80% of participants scored positive on at least one mental health-related domain.
- Considering the disturbingly high prevalence of mental health-related problem domains and high STI incidence, other interventions are needed to improve sexual and mental health.

10.1136/sextrans-2020-054438.supp2 Abstract translation This web only file has been produced by the BMJ Publishing Group from an electronic file supplied by the author(s) and has not been edited for content.
