# Supplementary material for: Enhancing help-seeking behaviour among men who have sex with men at risk for sexually transmitted infections: the syn.bas.in randomised controlled trial
Source: Sex Transm Infect. 2020 Jul 31;97(1):11–7. doi: 10.1136/sextrans-2020-054438 (PMC7841480; doi:10.1136/sextrans-2020-054438)
Supplement: Supplementary data [file sextrans-2020-054438supp001.pdf]

Appendix 1. Theoretical underpinning* of the health-seeking behavior intervention in the syndemic based intervention (syn.bas.in study) at the STI outpatient clinic in Amsterdam, 2016-2017.

Interventions	Definition	The syn.bas.in study
Education	Increasing knowledge or understanding	Providing insight in possible mental health related issues related to high risk behaviour
Persuasion	Using communication to induce positive or negative feelings or stimulate action	Based on questionnaire scores induce health seeking behaviour.
Incentivisation	Creating expectation of reward	Enquiring after actual health seeking behaviour after 12 months
Coercion	Creating expectation of punishment or cost	Not applicable and unwanted in an STI clinic setting where we strife for case holding
Training	Imparting skills	Not applicable in an STI clinic setting lacking required staff skills as far as any mental health interventions.
Restriction	Using rules to reduce the opportunity to engage in the target behaviour (or to increase the target behaviour by reducing the opportunity to engage in competing behaviours)	Not applicable and unwanted in an STI clinic setting where we strife for case holding
Environmental restructuring	Changing the physical or social context	Persons meeting the inclusion criteria actively approached to enter the study, via pop ups in the electronic patiënt file.
Modelling	Providing an example for people to aspire to or imitate	Not applied
Enablement	Increasing means/reducing barriers to increase capability or opportunity1.	On site opportunities to have intake consultations with substance use and mental health care providers.

*Definitions of interventions and policies. From: Michie S, van Stralen MM, West R. The behaviour change wheel: a new method for characterising and designing behaviour change interventions. *Implement Sci*. 2011;6:42. Published 2011 Apr 23. doi:10.1186/1748-5908-6-42

Appendix 2: Risk of bias table* of the health-seeking behavior intervention in the syndemic based intervention (syn.bas.in study) at the STI outpatient clinic in Amsterdam, 2016-2017.

Bias	Authors' judgment	Support for judgment
Random sequence generation (selection bias)	Low risk	Participants were assigned by block randomization in groups of 4, 6 or 8.
Allocation concealment (selection bias)	Low risk	Randomisation was performed with the assistance of a computer program.
Blinding of participants and researchers (performance bias)	High risk	Open label
Blinding of outcome assessment (detection bias)	High risk	Open label
Incomplete outcome data (attrition bias)	Low risk	Only 6 participants had outcome data that could not be ascertained. The proportion of missing data was not different between allocation arms.
Selective reporting (reporting bias)	Low risk	All prespecified outcomes were reported
Other bias	Low risk	Rates of loss to follow-up were similar between allocation arms.

*From: Higgins JP, Altman DG, Gøtzsche PC, et al. The Cochrane Collaboration's tool for assessing risk of bias in randomised trials. BMJ. 2011;343:d5928. Published 2011 Oct 18. doi:10.1136/bmj.d5928

Appendix 3: Study design of the health-seeking behavior intervention in the syndemic based intervention (syn.bas.in study) at the STI outpatient clinic in Amsterdam 2016-2017.

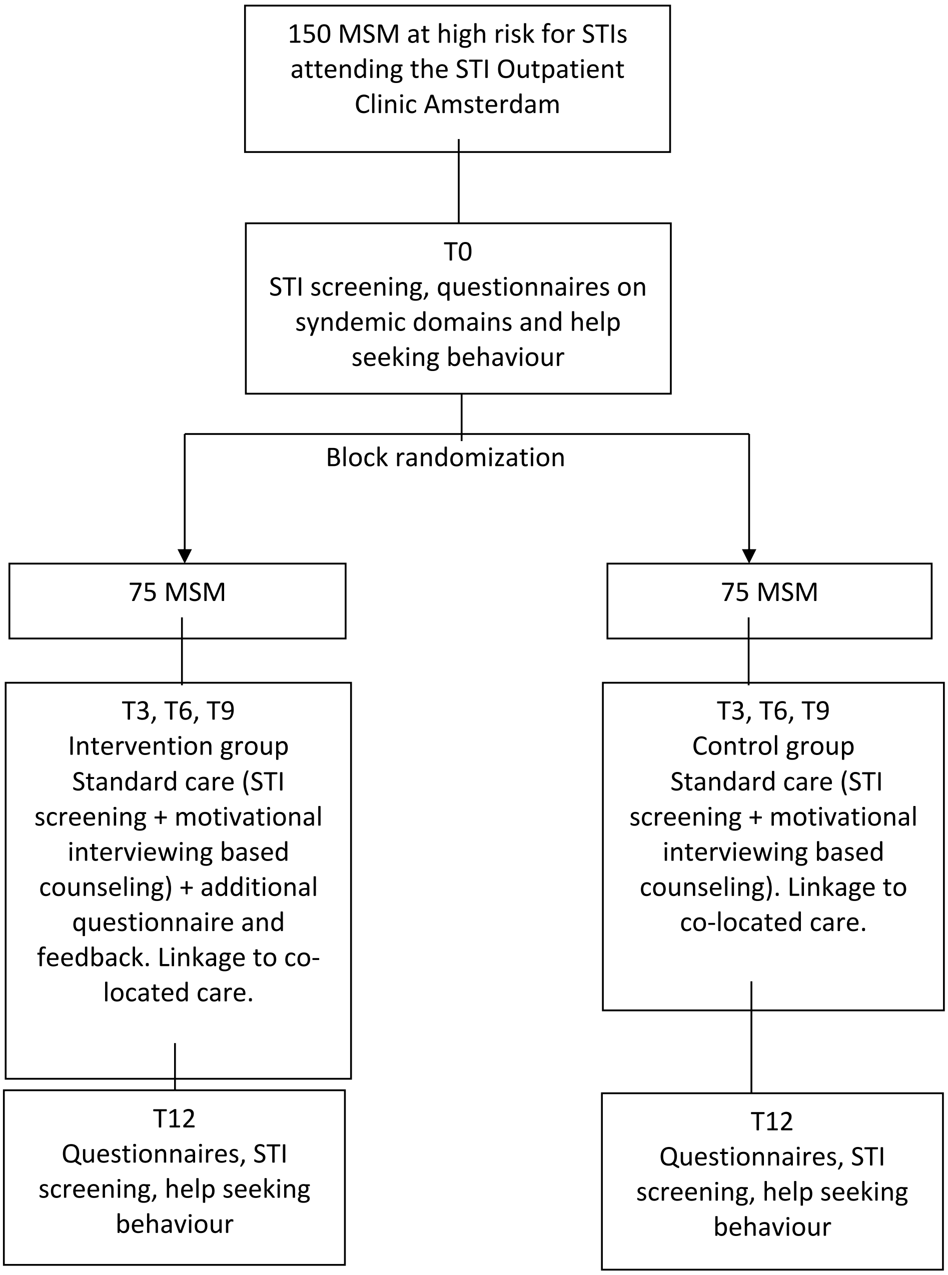

From: Achterbergh RCA, van der Helm JJ, van den Brink W, et al. Design of a syndemic based intervention to facilitate care for men who have sex with men with high risk behaviour: the syn.bas.in randomized controlled trial. *BMC Infect Dis* 2017;17(1):398. doi: 10.1186/s12879-017-2474-x.

Appendix 4. Substance use over time among men who have sex with men in the syndemic based intervention (syn.bas.in study) at the STI outpatient clinic in Amsterdam, 2016-2018.

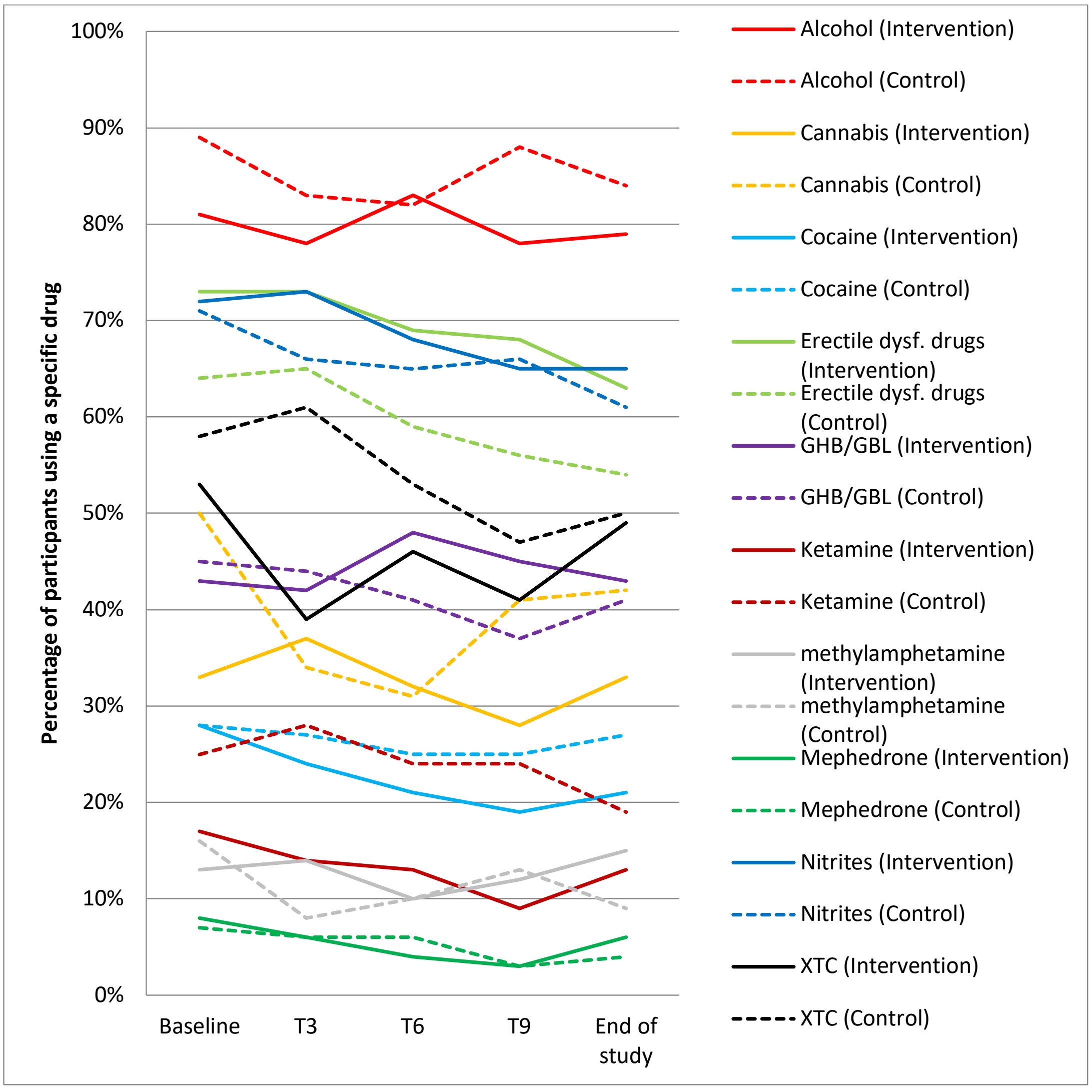

There were no significant differences between study groups, over time or in interaction between the two (*p*>0.05).

List of used abbreviations: STI: sexually transmitted infections; Dysf: dysfunction; GHB: gamma-hydroxybutyric acid; GBL: gamma-butyrolactone; XTC: Ecstasy;

Appendix 5. Univariable and multivariable analysis for confirmed help-seeking behaviour among 144 syn.bas.in participants (syn.bas.in study) at the STI clinic of Amsterdam, 2016-2018.

	Total (N=36/144)<br><br>n(%) within group	Uni-variable<br><br>OR [95% CI]	P-value	Multi-variable*<br><br>aOR [95% CI]	P-value
**Demographics**					
Age (years)			0.865		
<35	11/39 (28%)	1			
35-44	8/34 (24%)	0.7 [0.3-2.3]			
≥45	17/71 (24%)	0.8 [0.3-1.9]			
Country of origin **			0.062		0.044
Netherlands	28/94 (30%)	1		1	
Other	8/50 (16%)	0.4 [0.2-1.1]		0.4 [0.1-1.0]	
**Validated assessments on mental health related issues ***					
Harmful alcohol use	12/44 (27%)	1.2 [0.5-2.7]	0.678		
Drug related problems	24/74 (32%)	2.3 [1.1-5.1]	0.033		
Sexual compulsivity	13/24 (54%)	5.0 [2.0-12.5]	<0.001		
Depression	15/26 (58%)	6.3 [2.5-15.6]	0.001		
Anxiety	18/42 (43%)	3.5 [1.6-7.8]	0.002		
ADHD	8/13 (62%)	5.9 [1.8-19.4]	0.003		
Alexithymia	17/51 (33%)	1.9 [0.9-4.2]	0.091		
Partner violence (5y)	4/10 (40%)	2.1 [0.6-8.0]	0.279		
Childhood sexual abuse	8/16 (50%)	3.6 [1.2-10.4]	0.022		
Number of issues (per 1 increase) **		1.8 [1.4-2.2]	<0.001	1.9 [1.4-2.5]	<0.001
**Sexual health/behaviour characteristics**					
HIV status			0.685		
HIV negative	11/48 (23%)	1			
HIV positive	25/96 (26%)	1.2 [0.5-2.7]			
Ln of number of anal sex partners					
Per 1 increase		1.3 [0.9-1.9]	0.239		
Ln of number of condomless anal sex acts with casual partners **					
Per 1 increase		1.3 [0.9-1.9]	0.146		
Study group					
Control	16/72 (22%)	1	0.441		
Intervention	20/72 (28%)	1.3 [0.6-2.9]			

Analysis does not include the 6 individuals refusing consent to confirm help-seeking behaviour.
List of used abbreviations: STI: sexually transmitted infections; ADHD: attention deficit hyperactivity disorder; Ln: natural logarithm. * Due to multicollinearity between mental health issues, we only included number of issues (per 1 increase) in multivariable analysis. ** used in multivariable analysis.
