# Supplementary material for: Enhancing help-seeking behaviour among men who have sex with men at risk for sexually transmitted infections: the syn.bas.in randomised controlled trial
Source: Sex Transm Infect. 2020 Jul 31;97(1):11–7. doi: 10.1136/sextrans-2020-054438 (PMC7841480; doi:10.1136/sextrans-2020-054438)
Supplement: Abstract translation [file sextrans-2020-054438supp002.pdf]

Verbetering van hulpzoekgedrag onder mannen die seks hebben met mannen met veel seksueel overdraagbare aandoeningen: de syn.bas.in studie, een gerandomiseerde gecontroleerde trial

Inleiding:
Mannen die seks hebben met mannen (MSM) lopen een verhoogd risico op seksueel overdraagbare aandoeningen (soa's) en psychische stoornissen. De syndemie-theorie stelt dat psychosociale problemen gezamenlijk voorkomen en elkaar versterken, en dus seksueel risicogedrag vergroten. Het vaststellen, het verwijzen en vervolgen van psychosociale problemen zou het risicogedrag kunnen verminderen.

Methoden:
Bij de SOA poli van de GGD Amsterdam werden MSM geïncludeerd in de syn.bas.in studie, een op syndemie-gebaseerde, open-label gerandomiseerde, gecontroleerde interventie trial. We screenden deelnemers met gevalideerde vragenlijsten op de volgende probleemdomeinen: alcohol- en middelengebruik, seksuele compulsiviteit, angst, depressie, ADHD, alexithymie, intiem partnergeweld en seksueel misbruik bij kinderen. Deelnemers werden willekeurig toegewezen (1: 1) in 2 armen, om ofwel op maat gemaakte, persoonlijke feedback te krijgen om hulp te zoeken bij het screenen van geestelijke gezondheid, ofwel geen feedback en geen hulp advies te krijgen. Deelnemers werden een jaar lang driemaandelijks gevolgd. De primaire uitkomsten waren zelfgerapporteerd en bevestigd hulpzoekgedrag.

Resultaten:
We includeerden 155 MSM: 76 in de interventiegroep en 79 in de controlegroep. Bij inclusie scoorden 128 deelnemers (83,1%) positief in ten minste één probleemdomein. We vonden geen significant verschil in zelfgerapporteerd of bevestigd hulpzoekgedrag tussen de interventie en de controlegroep: respectievelijk 41% versus 29% (p = 0,14) en 28% versus 22% (p = 0,44). Er waren ook geen verschillen in SOA-incidentie en condoomloze anale seks tussen de twee groepen.

Conclusie:
Screening toonde een hoge prevalentie van problemen samenhangend met geestelijke gezondheid en middelengebruik. Feedback op maat, advies en verwijzing verhoogden het hulpzoekgedrag niet significant. Andere interventies zijn nodig om de hoge last van psychische stoornissen onder MSM aan te pakken.

Trial registratienummer NCT02859935.
